# Deviant Peer Affiliation and Non-Suicidal Self-Injury among Chinese Adolescents: Depression as a Mediator and Sensation Seeking as a Moderator

**DOI:** 10.3390/ijerph18168355

**Published:** 2021-08-06

**Authors:** Chang Wei, Jingjing Li, Chengfu Yu, Yanhan Chen, Shuangju Zhen, Wei Zhang

**Affiliations:** 1 Center for Studies of Psychological Application, School of Psychology, South China Normal University, Guangzhou 510631, China; changwei@m.scnu.edu.cn (C.W.); 2020010222@m.scnu.edu.cn (J.L.); 2019022970@m.scnu.edu.cn (Y.C.); shuangjuzhen@foxmail.com (S.Z.); 2 Research Center of Adolescent Psychology and Behavior, Department of Psychology, School of Education, Guangzhou University, Guangzhou 510006, China; yuchengfu@gzhu.edu.cn

**Keywords:** deviant peer affiliation, sensation seeking, depression, non-suicidal self-injury

## Abstract

Non-suicidal self-injury (NSSI) is an emerging health problem among adolescents. Although previous studies have shown that deviant peer affiliation is an important risk factor for this behavior, the reasons for this relationship are unclear. Based on the integrated theoretical model of the development and maintenance of NSSI and the social development model of delinquency prevention, this study tested whether depression mediated the relationship between deviant peer affiliation and NSSI and whether this mediating effect was moderated by sensation seeking. A sample of 854 Chinese adolescents (31.50% male; *M_age_* = 16.35; *SD* = 1.15) anonymously completed questionnaires on the study variables. Results of regression-based analyses showed that depression mediated the association between deviant peer affiliation and NSSI, and this effect was stronger among adolescents who reported high sensation seeking. The results demonstrate the role of individual differences in the link between affiliation with deviant peers and NSSI, and have implications for preventing and treating this risky behavior.

## 1. Introduction

Non-suicidal self-injury (NSSI) is the deliberate and direct destruction of one’s body tissue without suicidal intention [1]. One meta-analysis demonstrated that around 17.2% of adolescents in non-clinical samples reported NSSI [2]. NSSI is more prevalent among adolescents than children and adults [1,3]. Previous studies have shown that adolescent NSSI is not only associated with internalization problems [4,5] and externalization problems [6,7] but is also a key risk factor for future suicidal behaviors [8,9,10]. For example, in a longitudinal study, Guan et al. [9] found that NSSI was significantly positively associated with suicidal ideation and suicide attempts among adolescents. Therefore, it is important to identify the factors that are associated with an increased risk of adolescent NSSI.

Previous research showed that deviant peer affiliation is strongly correlated with risky behavior [11,12,13]. Deviant peer affiliation refers to selective affiliation with peers who engage in behaviors such as aggression, cheating, and substance abuse [14,15]. Deviant peer affiliation may also create a higher risk for NSSI. According to the social learning hypothesis of NSSI [1], adolescents may learn to engage in this risky behavior by observing their friends’ behavior. In line with this theory, deviant peer affiliation has been shown to be related to a higher likelihood of adolescent NSSI [16,17]. The above theory and empirical research results highlight the possibility that deviant peer affiliation is a potential risk factor for adolescent NSSI.

Although researchers have begun to focus on the link between deviant peer affiliation and adolescent NSSI, the underlying mediating and moderating mechanisms remain largely unexplored. Therefore, based on the integrated theoretical model of the development and maintenance of NSSI [18] and the social development model of delinquency prevention [19], the current research tested depression as a mediator and sensation seeking as a moderator of the relationship between deviant peer affiliation and adolescent NSSI.

### 1.1. Depression as a Potential Mediator

Depression is an emotional disorder which is thought be caused in part by an inability to cope with stressful events [20]. Depression has been shown to be a key risk factor associated with adolescent NSSI [21]. According to the integrated model of NSSI [18], risk factors (such as deviant peer affiliations) can lead to individuals’ aversive emotions (such as depression) and individuals engage in NSSI in order to immediately regulate aversive emotional experiences. Previous research also demonstrated that depression could mediate the association between peer relations and NSSI. For example, in a longitudinal study, Wu et al. [22] found that depression mediated the relationship between peer acceptance and lower NSSI in adolescents. Similarly, based on the multiple disadvantage model, Cheng and Li [23] tested the relationship between deviant peer affiliation, depression, and adolescent’ delinquency, and found that deviant peer affiliation may increase adolescents’ depression, which in turn increase their delinquency behaviors.

Evidence of the first part of the proposed mediation process (deviant peer affiliation → depression) comes from research showing that adolescents with deviant peer affiliation are more likely to be depressed [24,25]. This is because deviant peer affiliation may lead to an increase in externalizing problem behaviors, and the negative consequences of these problem behaviors may lead to an increase in internalizing problems such as depression [15]. For example, Li et al. [24] found that deviant peer affiliation was positively correlated with depression among adolescents. Similarly, Wang et al. [25] found that deviant peer affiliation was positively correlated with depressive symptoms among Chinese adolescents.

The second part of the proposed mediation process (depression → NSSI) can be conceptualized in terms of NSSI’s role in reducing aversive emotional states, including depression [26]. NSSI provides an immediate reduction in negative emotion, and is thus maintained through negative reinforcement. Several studies have shown that depression was positively correlated with adolescent NSSI [16,22]. Two longitudinal studies have also shown a link between depression and later NSSI. Wu et al. [22] found that adolescents’ depressive symptoms at Time 1 positively predicted NSSI at Time 2. Similarly, Prinstein et al. [16] found that depressive symptoms at Time 1 were positively correlated with NSSI at Time 2 and Time 3 among adolescents. Thus, based on theory and empirical research, it is reasonable for us to expect that deviant peer affiliation could increase adolescent NSSI via depression.

### 1.2. Sensation Seeking as a Moderator

Not all adolescents are equally harmed by deviant peer affiliation. Therefore, it is likely that there are variables that moderate the relationship between deviant peer affiliation and adolescent NSSI. Previous empirical research has found that sensation seeking is an important risk predictor of NSSI [27,28]. Sensation seeking refers to a personality trait characterized by a willingness to take risks in response to challenges, such as novel and complex experiences and strong emotions [29]. According to the social development model of delinquency prevention [19], the development of antisocial behavior (such as NSSI) is determined by the interaction of individual risk factors (such as sensation seeking and depression), and social environmental risk factors (such as deviant peer affiliation).

Sensation seeking, as a key individual difference, may strengthen the association between deviant peer affiliation and NSSI. Prior research elucidated that sensation seeking could moderate the relationship between a risky environment and antisocial behaviors [30,31,32]. For instance, sensation seeking interacted with deviant peer affiliation to predict drug use in a sample of Chinese reform school students [33]. Similarly, Yu et al. [32] found that adolescents’ high sensation seeking amplified the effect of cyberbullying victimization on NSSI.

There is also evidence that sensation seeking could be an important influence on the mediation process of interest in the current study. With regard to the first part of the mediation process, Xu [34] showed that sensation seeking interacted with negative interpersonal events as an environmental risk factor to predict adolescent depression. With regard to the second part of the mediation process, Karyadi et al. [35] found that college students’ sensation seeking interacted with labile anxiety/depression to predict adolescent risky behavior, namely hazardous alcohol use. In the current research, we tested the moderating role of sensation seeking in the developmental path from deviant peer affiliation to NSSI through depression.

### 1.3. The Present Study

**Figure 1:**
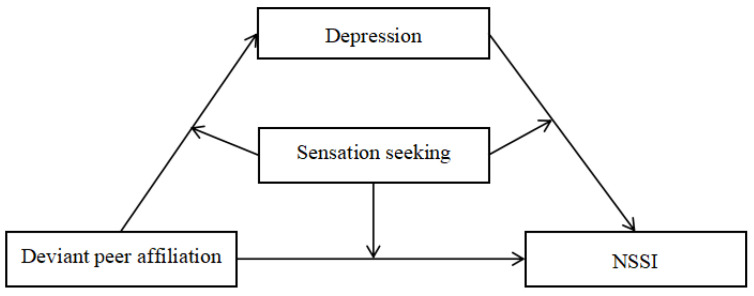
The proposed mediated moderation model. *Note:* NSS = non-suicidal self-injury.

On the basis of the integrated theoretical model of the development and maintenance of NSSI [18] and the social development model of delinquency prevention [19], we tested a moderated mediation model in which adolescents’ sensation seeking moderates the indirect relationship between deviant peer affiliation and adolescent NSSI through depression (see Figure 1). Specifically, we proposed the following hypotheses: (1) depression will mediate the association between deviant peer affiliation and NSSI; and (2) sensation seeking will moderate the indirect relation between deviant peer affiliation and NSSI, such that higher sensation seeking will intensify the indirect impact of deviant peer affiliation on NSSI.

## 2. Method

### 2.1. Participants

Participants were recruited from a secondary vocational and technical school in Hubei Province, central China, through random cluster sampling. A total of 854 adolescents (31.50% males, *n* = 269) participated in this study, all participants obtained informed consent from their parents, and none of them withdrew from the study. The average age was 16.35 years (*SD* = 1.15). Reflecting the demographics of the sample, 74.70% of participants’ mothers and 67.40% of their fathers had not completed high school or a secondary vocational and technical school.

### 2.2. Measures

#### 2.2.1. Deviant Peer Affiliation

Deviant peer affiliation was measured with the Deviant Peer Affiliation Scale [36], a Chinese language measure that includes 16 items (such as their friends “fighting”, “truancy or truancy”). This questionnaire asked participants to report how many of their friends expressed deviant behaviors in the past one week. Participants rated each item on a Likert score (1 = none to 5 = six or more). The responses were averaged across the 16 items, with higher scores indicating higher deviant peer affiliation. The scale was shown to have good reliability in a sample of Chinese adolescents [37]. Cronbach’s alpha in this study was 0.87.

#### 2.2.2. Depression

Depression was measured with the Chinese version [38] of the Center for Epidemiological Studies-Depression Scale (CES-D) [39]. Participants were asked to rate how often during the past one week they experienced different signs of depression, such as “I feel down” and “I feel lonely”. There are 20 items on the scale, Participants rated on a Likert scale (1 = less than one day to 4 = five to seven days). The responses were averaged across the 20 items, with higher scores indicating higher depression. Previous research showed that the 20-item scale had good reliability in a sample of Chinese adolescents [40]. Cronbach’s alpha in this study was 0.78.

#### 2.2.3. Sensation Seeking

Sensation seeking was measured with a short form [41] of the Sensation Seeking Scale [42]. This questionnaire includes six items (such as “I like to have new and exciting experiences even if they are a little frightening”), and each item was rated on a Likert scale (1 = almost always untrue to 6 = almost always true). The responses were averaged across the six items, with higher scores indicating a higher level of sensation seeking. The scale has demonstrated good reliability and validity in Chinese samples [43,44]. Cronbach’s alpha in this study was 0.70.

#### 2.2.4. NSSI

NSSI was measured with the Non-Suicidal Self-Injury Scale (NSSI) [45], which assesses the occurrence of seven behaviors including cutting, burning, and biting oneself. Each item was scored on 4-point scale (1 = never to 4 = six or more times). The responses were averaged across the 7 items, with higher scores indicating more frequent NSSI. Previous research showed that the scale had good reliability in measuring Chinese adolescents’ NSSI [32]. Cronbach’s alpha in this study was 0.84.

#### 2.2.5. Control Variables

Compared to males, females have been shown to engage in more NSSI [46], and NSSI is correlated with age in youth samples [47]. Therefore, we controlled for gender and age in the statistical analyses. Gender was a dichotomous variable (1 = male; 0 = female).

### 2.3. Procedure and Statistical Analyses

This research was approved by the Ethics in Human Research Committee of the Department of Psychology, Guangzhou University. The data were collected in the respondents’ classes by well-trained college students and teachers. We obtained informed consent from adolescents, parents, and teachers before collecting data. In addition, we informed adolescents that they were free to withdraw from the study test at any time. All the data collected are anonymous and only used for scientific research purposes.

SPSS 21.0 was used to generate descriptive statistics and correlations. Mediation and moderation effects were tested with Mplus 8.3. Missing data were handled via the FIML estimation method. We used bootstrapping with 2000 iterations to verify the significance of the paths in the model. According to convention, the model fit is considered good when *χ*^2^/*df* < 5, CFI > 0.90, TLI > 0.90, RMSEA < 0.08, and SRMR < 0.08 (Hoyle, 2012).

## 3. Results

### 3.1. Preliminary Analyses

The means, standard deviations, and correlation coefficients for all research variables are displayed in Table 1. The results indicated that deviant peer affiliation, sensation seeking, and depression were all positively correlated with NSSI. Deviant peer affiliation was also correlated with sensation seeking and depression.

### 3.2. Mediation Effect of Deviant Peer Affiliation

**Figure 2:**
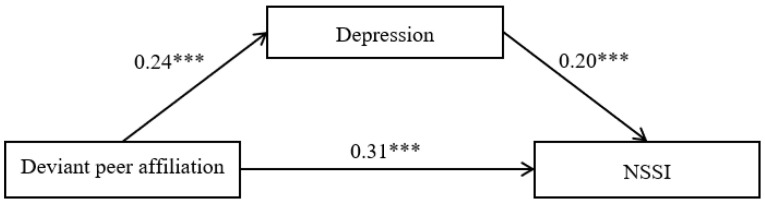
Model of the mediating role of depression in the association between deviant peer affiliation and non-suicidal self-injury. Note: the numbers are standardized regression coefficients. Path coefficients between control variables (gender and age) and each of the variables in the model are not displayed. Gender was dummy coded as 1 = male, 0 = female. Of those paths, gender was significantly related to depression (*b* = −0.14, *SE* = 0.04, *t* = −4.13, *p* < 0.001) and NSSI (*b* = −0.08, *SE* = 0.04, *t* = −2.08, *p* < 0.05). NSSI = non-suicidal self-injury. *** *p* < 0.001.

The hypothesized mediation model showed a good fit to the data, *χ*^2^/*df* = 2.37, CFI = 0.99, RMSEA = 0.04, and SRMR = 0.02. Figure 2 displays the paths in the proposed model. Deviant peer affiliation positively predicted depression (*b* = 0.24, *SE* = 0.05, *t* = 5.29, *p* < 0.001) and NSSI (*b* = 0.31, *SE* = 0.06, *t* = 4.85, *p* < 0.001), and depression positively predicted NSSI (*b* = 0.20, *SE* = 0.03, *t* = 6.13, *p* < 0.001). Furthermore, bootstrapping analyses showed that depression partially mediated the pathway from deviant peer affiliation to NSSI (indirect effect = 0.05, *SE* = 0.01, 95% CI = [0.03, 0.07]).

### 3.3. Moderated Mediation

**Figure 3:**
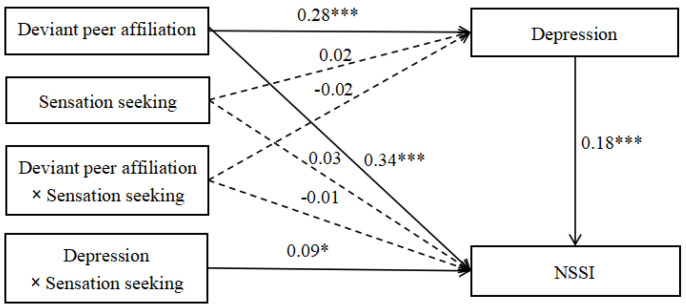
Model of the moderating role of sensation seeking on the indirect relationship between deviant peer affiliation and NSSI, Note: The numbers are standardized regression coefficients. Path coefficients between control variables (gender and age) and each of the variables in the model are not displayed. Of those paths, gender was significantly associated with depression (*b* = −0.15, *SE* = 0.04, *t* = −4.09, *p* < 0.001). NSSI = non−suicidal self−injury. * *p* < 0.05; *** *p* < 0.001.

The moderated mediation model represented in Figure 3 revealed a good fit to the data: *χ*^2^/*df* = 2.74, CFI = 0.99, RMSEA = 0.05, and SRMR = 0.02. Deviant peer affiliation (*b* = 0.34, *SE* = 0.08, *t* = 4.31, *p* < 0.001) and depression (*b* = 0.18, *SE* = 0.03, *t* = 5.29, *p* < 0.001) were significantly associated with NSSI. Deviant peer affiliation was significantly associated with depression (*b* = 0.28, *SE* = 0.05, *t* = 5.95, *p* < 0.001), but sensation seeking was not significantly associated with depression (*b* = 0.02, *SE* = 0.04, *t* = 0.44, *p* > 0.05) or NSSI (*b* = 0.03, *SE* = 0.04, *t* = 0.65, *p* > 0.05). More importantly, sensation seeking significantly moderated the impact of depression on NSSI (*b* = 0.09, *SE* = 0.04, *t* = 2.26, *p* < 0.05).

**Figure 4:**
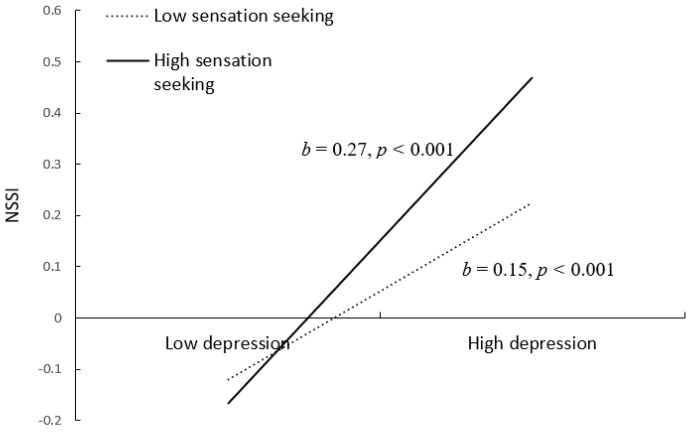
Interactive effect of depression and sensation seeking on NSSI. Note: Sensation seeking is graphed for two levels: high sensation seeking (1 SD above the mean) and low sensation seeking (1 SD below the mean). NSSI = nonsuicidal self−injury. NSSI = non-suicidal self−injury.

We conducted simple slope tests to better understand the results regarding sensation seeking as a moderator. As depicted in Figure 4, when youth showed higher sensation seeking, the relation between depression and NSSI was significant (*b* = 0.27, *SE* = 0.04, *t* = 7.41, *p* < 0.001). However, when youth showed lower sensation seeking, this relation was weaker, although still statistically significant (*b* = 0.15, *SE* = 0.04, *t* = 3.64, *p* < 0.001). Therefore, there was partial evidence that the mediating effect of depression in the association between deviant peer affiliation and adolescent NSSI was stronger in youth who were high sensation seekers.

## 4. Discussion

Despite deviant peer affiliation having been identified as a risk factor for adolescents’ NSSI [11,16,17], no research to date has examined the mechanisms of this effect or factors that might ameliorate or exacerbate it. Guided by the integrated theoretical model of the development and maintenance of NSSI [18] and the social development model of delinquency prevention [19], this study tested whether depression mediated the relationship between deviant peer affiliation and NSSI, and whether this mediating effect was moderated by youth’s sensation seeking. As expected, we found that depression mediated the association between deviant peer affiliation and NSSI, and the indirect effect of depression was strengthened by sensation seeking.

### 4.1. The Mediating Role of Depression

Consistent with our hypothesis, we found that depression mediated the relationship between deviant peer affiliation and NSSI in a non-clinical sample of adolescents. This shows that depression is a key bridge linking deviant peer affiliation to NSSI in this developmental period. In other words, deviant peer affiliation may lead to adolescents’ depression, which, in turn, could increase antisocial behaviors such as NSSI. Therefore, depression is a possible explanatory mechanism that might explain why adolescents with deviant peer affiliation are more likely to engage in NSSI. Our results are consistent with those of other studies that have reported that deviant peer affiliation and depression increased the risk of engaging NSSI [17,21]. Depression has also been found to mediate the relationship between peer acceptance and NSSI [22].

Furthermore, each link in this mediation model is worth discussing. Tests of the first stage (i.e., deviant peer affiliation → depression) found that deviant peer affiliation was positively associated with depression among adolescents. Our finding is consistent with prior studies showing that deviant peer affiliation was significantly associated with depression [24]. One potential explanation is that deviant peer affiliation may lead to externalizing problems [31], which, in turn, would increase the probability of internalizing problems among these youth. Additionally, this study found that deviant peer affiliation is an important risk factor for depression among adolescents. Previous empirical studies mainly focused on the relationship between deviant peer affiliation and adolescents’ externalized behaviors such as online game addiction, tobacco use, and alcohol use [13,31]. Only a few studies [24,48] have examined the relationship between deviant peer affiliation and internalizing. Therefore, this study enriches the literature on this topic.

The results also provided evidence of the second stage of the proposed mediation process (i.e., depression → NSSI), consistent with prior research [16,22]. The function theory of NSSI would suggest that NSSI as a maladaptive coping strategy has the function of automatic negative reinforcement [1], and adolescents may use NSSI to get temporary relief from their feelings of depression.

Finally, in addition to finding evidence of both parts of the proposed mediation model, the results also showed that adolescent depression significantly mediated the link between deviant peer affiliation and NSSI. This finding further deepens and enriches the findings from a small number of studies that tested this association [16,17]. In addition, this research provides a basis for intervention to prevent and reduce adolescent NSSI. For instance, the results showed that deviant peer affiliation and depression were important risk factors for adolescents’ NSSI. Therefore, intervention measures might reduce adolescent NSSI by intervening to reduce these two risk factors.

### 4.2. The Moderating Role of Sensation Seeking

In the current study, sensation seeking strengthened the mediation effect of depression in the relationship between deviant peer affiliation and NSSI. These results were consistent with our hypothesis and with the social development model of delinquency prevention [19]. Further analysis was conducted to determine what part of the mediation process was moderated by sensation seeking. Follow-up analyses suggested that sensation seeking conferred a risk effect in the second part of the indirect pathway. To be specific, high levels of sensation seeking significantly amplified the impact of depression on adolescent NSSI. These findings were in alignment with a prior study that found that sensation seeking magnified the association between a risk factor (cyberbullying victimization) and adolescent NSSI [32]. According to the sensation seeking theory [49,50], individuals who seek high sensation also tend to enjoy novel experiences and taking risks, which in turn increases the possibility that they will use a risky behavior such as NSSI to escape depression [51]. Thus, for adolescents with high levels of sensation seeking, the relation between depression and NSSI became stronger.

Contrary to our speculation, the direct relationship between deviant peer affiliation and NSSI was not moderated by sensation seeking. The relationship between deviant peer affiliation and depression was also not moderated by sensation seeking. In other words, deviant peer affiliation was always positively associated with depression and NSSI among adolescents, regardless of the level of sensation seeking. Such a finding highlights the risk role of deviant peer affiliation in the development of depression and NSSI among adolescents. With the growth of age, adolescents spend more time in school [52] and interact more with their peers, so peers have more influence on adolescents [53]. In addition, the role of peers becomes more important during adolescence [54]. Thus, adolescents are more strongly influenced by peers, regardless of the level of sensation seeking. This result supports the view that peers have a crucial impact on adolescent development [22,32,48].

Overall, our study constructs a moderated mediation model for exploring “how and for whom” potential risk factors take affected adolescent NSSI. Specifically, high levels of sensation seeking amplified the negative impact of deviant peer affiliation on NSSI via depression. These results underscore the important roles of sensation seeking and depression in understanding the link between deviant peer affiliation and NSSI among Chinese adolescents.

### 4.3. Limitations and Future Directions

First, this research was performed using a cross-sectional study design, making it impossible to determine causality and reverse causality. For instance, adolescents who engage in NSSI may have difficulty with communicating and interacting with their typical peer group, in turn increasing interactions with deviant peers. Future studies should employ a longitudinal design with multiple time points to explore the causal relationship between variables. Second, the data in the current study were self-reports by adolescents, and thus the results could be affected by common method bias. Future studies can use multiple informants (such as friends’ reports and teachers’ reports) to collect data. Third, future research could test other mediating variables (such as school engagement; Yu et al. [32] and moderating variables (such as rumination [55]) to further explore the relationship between deviant peer affiliation and NSSI among adolescents. Fourth, the study was conducted in mainland China, and further research can test whether the results can be generalized to other cultural contexts.

## 5. Conclusions

The results showed that depression mediated the relationship between deviant peer affiliation and NSSI among adolescents. In addition, sensation seeking strengthened the mediation effect of depression in the relationship between deviant peer affiliation and NSSI (the second part of mediation process).

## Figures and Tables

**Table 1:** Descriptive statistics and ceorrelations for all variables.

Variable	1	2	3	4	5	6
1. Gender	1.00					
2. Age	0.07 *	1.00				
3. DPA	0.19 ***	0.00	1.00			
4. SS	0.01	−0.06	0.16 ***	1.00		
5. Depression	−0.10 **	−0.06	0.22 ***	0.06	1.00	
6. NSSI	−0.04	−0.05	0.35 ***	0.10 **	0.29 ***	1.00
*Mean*	0.31	16.35	1.33	3.11	1.81	1.10
*SD*	0.47	1.15	0.45	0.97	0.36	0.27

Note: *N* = 854. Gender was dummy coded as 1 = male, 0 = female. DPA = deviant peer affiliation; SS = sensation seeking; NSSI = non-suicidal self-injury. * *p* < 0.05, ** *p* < 0.01, *** *p* < 0.001.

## Data Availability

The data presented in this study are available on request from the corresponding author.
